# Environmental and Household Sources Associated with Elevated Blood Lead Levels Among Mexican Women of Childbearing Age: A Community-Based Screening Study

**DOI:** 10.3390/toxics14080695

**Published:** 2026-08-06

**Authors:** Larissa Betanzos-Robledo, Guadalupe Estrada-Gutierrez, Hector Lamadrid-Figueroa, Karen Vázquez-Gutiérrez, Martha María Téllez-Rojo, Manish Arora, Alejandra Cantoral

**Affiliations:** 1 Doctoral Program in Medical Sciences, National Autonomous University of Mexico, Mexico City 04510, Mexico; larissa.betanzos@comunidad.unam.mx; 2 Department of Immunobiochemistry, National Institute of Perinatology, Mexico City 11000, Mexico; guadalupe.estrada@inper.gob.mx; 3 Department of Perinatal Health, Center for Population Health Research, National Institute of Public Health, Cuernavaca 62100, Mexico; hlamadrid@insp.mx; 4 Centro Ibero Meneses, Iberoamericana University, Mexico City 01210, Mexico; karen.vazquez@centromeneses.mx; 5 Center for Nutrition and Health Research, National Institute of Public Health, Cuernavaca 62100, Mexico; mmtellez@insp.mx; 6 Department of Environmental Medicine and Public Health, Icahn School of Medicine, Mount Sinai, NY 10029, USA; manish.arora@mssm.edu; 7 Health Department, Iberoamericana University, Mexico City 01376, Mexico

**Keywords:** lead exposure, environmental exposure, elevated blood lead levels

## Abstract

Background: Lead (Pb) exposure remains a significant public health concern, particularly among women of childbearing age, because of its adverse effects on maternal–child health. Although lead-glazed ceramics (LGC) for cooking or storing food are a well-recognized source of Pb exposure in Mexico, other environmental and household sources remain insufficiently characterized. Objective: To identify environmental and household factors associated with elevated blood lead levels (BLLs) among women of childbearing age participating in a community-based screening in Mexico City. Methods: We conducted a cross-sectional analysis of 158 women aged 18–40 years. Capillary BLLs were measured using the LeadCare^®^ II system. Participants completed a structured questionnaire assessing potential Pb sources of exposure. Elevated BLL was defined as ≥3.5 µg/dL. Associations between exposure sources and elevated BLL were evaluated using multivariable logistic regression adjusted for sociodemographic and lifestyle factors. Shapley decomposition was used to estimate the relative contribution of each exposure source to the model. Results: The prevalence of elevated BLLs was 31%. Women reporting both past and current use of LGC had higher odds of elevated BLLs than women who had never used LGC (adjusted OR = 9.43; 95% CI: 1.85–48.15). Living in or regularly visiting a recently remodeled home (adjusted OR = 3.21; 95% CI: 1.45–7.11) and living near a highway (adjusted OR = 2.41; 95% CI: 1.07–5.48) were also independently associated with elevated BLLs. LGC use was the largest contributor to the model performance (47.46%), followed by home remodeling (31.78%) and proximity to highways (20.76%). Conclusions: Our findings support LGC as an important source of Pb exposure while identify home remodeling and residential proximity to highways as additional sources associated with elevated BLLs. Together, these findings broaden the understanding of Pb exposure pathways among women of childbearing age and support the need for prevention and screening strategies.

## 1. Introduction

Lead (Pb) is a heavy metal recognized as a major public health concern due to its well-documented toxic effects on human health [1]. Although Pb exposure affects individuals of all ages, women of childbearing age represent a particularly vulnerable group, as chronic exposure may have long-term consequences for both maternal health and offspring development [2,3]. Pb exposure throughout the life course occurs primarily through ingestion or inhalation and, in adults, approximately 95% of the total body burden is stored in bone, where it can persist for decades [4].

Pb is a multisystem toxicant with no known safe level of exposure. In adults, chronic Pb exposure has been associated with hypertension, renal dysfunction, and adverse reproductive outcomes [5]. During pregnancy, especially in the second and third trimesters, Pb stored in maternal bone can be mobilized and cross the placenta, increasing fetal exposure [6,7] and contributing to an increased risk of adverse pregnancy outcomes, including spontaneous abortion [8], preterm delivery [9], low birth weight [10], preeclampsia [11,12], and long-term effects on child health, particularly impaired cognitive development [13]. Prenatal and early-life lead exposure is of particular concern because it can adversely affect the developing nervous system, resulting in reduced IQ, impaired cognitive function, behavioral disorders, attention deficits, and long-term neurodevelopmental impairment [14,15,16]. These health effects highlight the importance of identifying and reducing Pb exposure among women of reproductive age before and during pregnancy.

There is no safe level of Pb exposure, as even low blood lead levels (BLLs) have been associated with irreversible adverse health effects [17]. Reflecting the accumulating evidence that adverse effects occur at progressively lower BLLs, the U.S. Centers for Disease Control and Prevention (CDC) lowered the BLLs reference value for children to 3.5 µg/dL in 2021 [18]. Subsequently, the Council of State and Territorial Epidemiologists (CSTE) recommended adopting the same threshold for adults to support surveillance and early identification of elevated Pb exposure [19,20]. In Mexico, national guidelines currently establish an action level of 5 µg/dL for children under 15 years of age and for pregnant and breastfeeding women [21].

In Mexico, the use of lead-glazed ceramics (LGC) for cooking, serving, and storing food has been consistently identified as the primary source of Pb exposure in the general population [22]. Pb leaching from these ceramics is mainly attributed to the traditional glazing process, which uses lead-based glazes and often involves firing temperatures insufficient to fully vitrify the glaze, leaving bioavailable Pb on the ceramic surface. During household use, repeated heating, glaze deterioration, and contact with acidic foods, such as tomato-based sauces and citrus fruits, further promote Pb leaching into food and beverages [23]. In Mexico, the safety of LGC is regulated by NOM-231-SSA1-2016, which establishes maximum permissible limits for Pb and standardized testing procedures to evaluate their leaching from food-contact materials [24]. Despite these regulations, artisanal LGC remains widely used, because of its cultural, culinary, and economic importance [25].

Although LGC is well established as an important source of Pb exposure in the Mexican population, evidence from national surveys suggests that it accounts for only approximately half of elevated BLLs cases, indicating that additional sources remain to be identified [26].

These additional sources may be particularly relevant in Mexico City, where decades of intense vehicular traffic before the phase-out of leaded gasoline contributed to widespread Pb accumulation in roadside soils and urban dust, and where aging housing infrastructure may further increase residential exposure. These environmental and household factors may represent important but less characterized sources of Pb exposure, underscoring the importance of evaluating multiple environmental pathways when assessing Pb exposure in urban populations [27,28,29].

Identifying these sources is essential for the development of targeted prevention strategies aimed at reducing Pb exposure among women of childbearing age and mitigating adverse maternal and offspring health outcomes. Therefore, the objective of this study was to identify residential and household factors associated with elevated blood lead levels in women of childbearing age participating in a community-based screening program in Mexico City.

## 2. Material and Methods

### 2.1. Study Population

The present analysis was conducted in a sample of 158 women of childbearing age participating in a community-based screening protocol. An open invitation to participate was extended during October and November 2022 through the Iberoamericana University Community Center’s social media and community outreach activities in nearby neighborhoods, to women who met the following inclusion criteria: age between 18 and 40 years, residence in the Santa Fe community in the Álvaro Obregón municipality of Mexico City, not being pregnant or breastfeeding at the time of enrollment and having provided written informed consent. Participation was voluntary and based on convenience sampling rather than a representative sample of women of reproductive age in Mexico City.

The investigations were conducted in accordance with the principles outlined in the Declaration of Helsinki (1975, revised in 2013). All participants provided written informed consent prior to participation. The study protocol was approved by the Institutional Review Board of Iberoamericana University (approval ID: 3/22).

Screening procedures aimed at identifying women with elevated blood lead levels were conducted at the Ibero Meneses Community Center facilities by trained and standardized personnel.

### 2.2. Blood Lead Levels (BLLs) Measurements

Capillary Blood Pb levels (BLLs) were quantified using the LeadCare^®^ II system (Magellan Diagnostics, North Billerica, MA, USA), which employs portable anodic stripping voltammetry, with an analytical detection range of 3.3–65 µg/dL. Approximately 50 µL of capillary blood (one drop) was obtained by finger puncture and mixed with the LeadCare^®^ II treatment reagent to release Pb from the blood matrix. Results were displayed on the device screen within approximately three minutes.

To minimize external contamination, finger puncture sites were prepared according to standardized protocol, blood collection was performed by trained personnel, and all samples were processed following quality assurance procedures including instrument calibration and internal quality control according to the manufacturer’s recommendations [30]. Measurements ≥25 µg/dL were repeated in accordance with the current Mexican Official Standard [21]. As part of the quality assurance procedures, BLLs were independently measured in a subsample of 47 participants using inductively coupled plasma mass spectrometry (ICP-MS) (Agilent 7800 ICP-MS, Agilent Technologies, Santa Clara, CA, USA), the reference analytical method for Pb determination in whole blood. Correlation between LeadCare^®^ II and ICP-MS measurements was assessed using Spearman’s rank correlation coefficient and agreement between the two methods was evaluated using Bland–Altman analysis.

### 2.3. Lead Exposure Questionnaire

Participants completed a Pb exposure screening questionnaire consisting of 15 items, adapted from questionnaires used in the National Health and Nutrition Survey and supplemented with additional items identified in the literature. Responses were recorded as dichotomous variables (yes/no). Potential Pb exposure sources were grouped into four categories: occupation or para-occupational sources, residential sources, use of lead glazed ceramics (LGC), and other exposure sources.

The questionnaire assessed the following sources of Pb exposure: living in or regularly visiting a recently remodeled home (no standardized definition of “recently remodeled” was provided) residence near (defined as a proximity of a four-block radius) of a pottery workshop, mine, polluted river, garbage dump, busy street or highway; proximity to radiator workshops, ironworks, foundries, textile factories, or printing presses; past (during childhood) and current (in the last months) use of LGC for cooking and/or storing food; preparation and storage of food in LGC (in the last week); use of traditional remedies greta, azarcon, or navajo clay for gastrointestinal symptoms; spending time with adults whose occupation or hobbies involve Pb exposure (e.g., painting or remodeling houses, welding, auto repair, battery manufacturing, glass or ceramics, fishing lures, shooting ranges); and personal or family history of lead poisoning. To better characterize participants’ history of exposure to LGC, a composite four-category variable was created by combining self-reported past use during childhood and current use during the previous month. Participants were classified as follows: (1) never use (no past or current use), (2) past use only (childhood use but no current use), (3) current use only (current use but no reported childhood use), and (4) past and current use (both childhood and current use). This composite variable was used to evaluate cumulative patterns of LGC use across the life course. This approach allowed us to distinguish between historical, recent, and persistent exposure patterns while reducing redundancy between correlated exposure variables.

These sources of Pb exposure were grouped into two main categories: 1. Environmental factors: Characteristics of the environment in which the person lives, including neighborhood and community-level sources of exposure, such as residential proximity to industrial activities, contaminated environments, and other external sources of lead exposure. 2. Household factors: Household-level practices and sources occurring within the home environment, including the use of lead-glazed pottery, occupational take-home exposure, and other indoor exposure sources.

### 2.4. Covariates

Participants completed a questionnaire on socioeconomic and lifestyle information, including age, education, marital status, smoking status and alcohol consumption. Weight and height were measured using a bioelectrical impedance analyzer (InBody 270, InBody Co., Ltd., Seoul, Republic of Korea), and body mass index (BMI, kg/m^2^) was calculated. Participants were classified as underweight (<18.5 kg/m^2^), normal weight (18.5–<25.0 kg/m^2^), overweight (25.0–<30.0 kg/m^2^), or as having obesity (≥30.0 kg/m^2^) [31].

Venous blood samples were collected by trained phlebotomists using standard aseptic techniques into EDTA tubes. Hemoglobin (Hb) concentration was determined in the clinical laboratory as part of a complete blood count using an automated hematology analyzer, following the manufacturer’s instructions and the laboratory’s internal quality control procedures. Hb values were adjusted for the altitude of Mexico City in accordance with World Health Organization recommendations and anemia was defined as Hb < 12 g/dL [32].

Dietary intake of calcium (Ca), iron (Fe), and zinc (Zn) was assessed using a 24 h dietary recall administered by a trained nutritionist [33], given the known association between micronutrient deficiencies and Pb absorption [5].

### 2.5. Statistical Analysis

Descriptive analyses were conducted to summarize the main characteristics of the study population. Categorical variables are presented as frequencies and proportions, and continuous variables as means or medians, according to their distribution. Participants were categorized according to capillary BLLs as having low BLLs (<3.5 µg/dL) or elevated BLLs (≥3.5 µg/dL) in accordance with the most up-to-date public health guidance, providing a more sensitive criterion for identifying women with elevated Pb exposure who may benefit from preventive interventions [19,20].

Differences in participant characteristics by BLLs category were evaluated using Fisher’s exact test for the categorical variables and Student’s *t*-test or Wilcoxon rank-sum test for continuous variables. Associations between reported Pb exposure sources and BLLs categories were initially examined using Fisher’s exact test. These bivariate analyses were performed as exploratory analyses to identify potential factors associated with elevated BLLs. Variables considered biologically plausible or supported by previous literature, together with those identified in the exploratory analyses, were evaluated in the multivariable logistic regression model.

Odds ratios (OR) and 95% confidence intervals (CI) for the association between Pb exposure sources and elevated BLLs were estimated using logistic regression models. Multivariable models were adjusted for age, educational attainment, and marital status. Finally, to further explore the relative contribution of each Pb source of exposure to the predictive performance of the multivariable logistic regression model, we conducted a Shapley pseudo-R^2^ decomposition analysis. Shapley decomposition is an approach which distributes the model’s explained variance among the predictor variables by estimating their average marginal contribution across all possible combinations of predictor variables. In this study, the decomposition was applied to the pseudo-R^2^ of the multivariable logistic regression model to assess the relative contribution of each source of exposure to the model’s overall performance.

All statistical analyses were conducted using Stata version 18 (StataCorp LLC, College Station, TX, USA), and statistical significance was defined as a two-sided *p*-value < 0.05.

## 3. Results

The main characteristics of the study population overall and according to BLLs categories are presented in Table 1. A total of 158 women of childbearing age were included in the analysis. The mean age was 29.7 ± 6.5 years. Most completed high school education (49%), and 57% were married or in a civil union. A total of 68% of participants reported alcohol consumption and 37% reported tobacco use. The combined prevalence of overweight and obesity was 63%, and 17% participants had Hb levels below 12 g/dL, indicating anemia. The mean (IQR) intakes of Ca, Fe and Zn were 795.4 mg (539.5–1143.1), 13.3 mg (10.2–17.2), and 11.5 mg (8.1–16.2), respectively. The overall prevalence of elevated BLLs (≥3.5 μg/dL) was 31% (n = 49). No statistically significant differences were found in general characteristics between participants with low and elevated BLLs.

Potential sources of Pb exposure according to BLLs categories are presented in Table 2. Among environmental factors evaluated, living near a highway was more common among women with elevated BLLs than those with low BLLs (78% vs. 58%, *p* = 0.020). Among household factors, women with elevated BLLs were more likely to report persistent use of lead-glazed ceramics (past and current use) compared with women with low BLLs (73% vs. 44%, *p* = 0.004). Likewise, living in or regularly visiting a recently remodeled home was more frequent among women with elevated BLLs (45% vs. 20%, *p* = 0.002). No significant differences were observed for the remaining exposure variables.

Logistic regression analyses of potential Pb exposure sources and elevated BLLs are presented in Table 3. Among the environmental factors evaluated, women who reported living near a highway had higher odds of elevated BLLs than those who did not (crude OR = 2.52; 95% CI: 1.17–5.45; *p* = 0.019). This association remained statistically significant after adjustment for sociodemographic and dietary factors (adjusted OR = 2.41; 95% CI: 1.07–5.48; *p* = 0.034).

Among household factors, persistent use of lead-glazed ceramics (past and current use) was strongly associated with elevated BLLs, with women reporting both past and current use having more than ninefold higher odds of elevated BLLs compared with those who had never used lead-glazed ceramics (adjusted OR = 9.43; 95% CI: 1.85–48.15; *p* = 0.007). Likewise, women who reported living in or regularly visiting a recently remodeled home had more than threefold higher odds of elevated BLLs (adjusted OR = 3.21; 95% CI: 1.45–7.11; *p* = 0.004). Results for all other potential Pb exposure sources evaluated in the questionnaire are presented in Supplementary Table S1. None of the remaining exposure variables was significantly associated with elevated BLLs.

Among all evaluated exposure sources, Shapley decomposition indicated that reported use of lead-glazed ceramics was the largest contributor to the model’s explanatory power (47.46% of the model pseudo-R^2^), followed by living in or regularly visiting a recently remodeled home (31.78%) and living near a highway (20.76%) (Table 4).

## 4. Discussion

In this study, we found that environmental and household factors, including use of LGC, living in or regularly visiting a recently remodeled home and living near a highway, were associated with high blood lead levels (BLLs) (≥3.5 µg/dL) among women of childbearing age (mentioned in descending order according to their relative predictive contribution in the multivariable model).

In our study, use of LGC was the household factor most strongly associated with elevated BLLs, supporting previous evidence that this traditional cookware remains a major source of Pb exposure among women of childbearing age in Mexico. Our findings are consistent with previous studies conducted in different regions of the country. At the national level, analyses of the Mexican National Health and Nutrition Survey (ENSANUT) have identified the use of LGC as one of the main determinants of elevated BLLs among children aged 1–4 years, highlighting its substantial contribution to lead poisoning in Mexico [22]. Similarly, a study conducted in Mexico City reported that the use of LGC for food preparation is associated with higher BLLs and exhibits a dose–response relationship with frequency of use [34]. Likewise, isotopic analyses have further confirmed LGC as a major source of Pb exposure by demonstrating strong correlations between Pb in blood and ceramic materials [35]. Similar associations have also been reported among pregnant women in Durango and other Mexican populations, where LGC users consistently exhibited higher BLLs than non-users [36]. Our questionnaire captured both childhood and recent use of LGC, allowing us to examine patterns of exposure across different stages of life. Interestingly, women who reported both past and current use of LGC had the highest odds of elevated BLLs, whereas reporting only past or only current use was not significantly associated with elevated BLLs after adjustment. These findings suggest that exposure to LGC across different stages of life may be a more informative indicator of Pb exposure than use restricted to a single period. The observed association among women reporting both childhood and current LGC use may reflect the cumulative effects of repeated external Pb exposure over time. Although bone represents the major reservoir of Pb in the human body and an endogenous contribution from skeletal Pb cannot be excluded, increased Pb mobilization has primarily been documented during periods of high bone turnover, such as pregnancy, lactation, and menopause. Because bone Pb was not measured in this study, the relative contribution of endogenous Pb release to circulating BLLs cannot be determined [5,37].

Our findings are consistent with previous evidence suggesting that despite longstanding recognition of this exposure source, lead-glazed pottery continues to represent a persistent public health problem in Mexico.

The biological mechanism underlying this association is well established. Lead-containing glazes can release Pb into food and beverages through leaching, particularly when LGCs are used to prepare or store acidic food or after prolonged heating. Pb leaching is influenced by several factors, including the Pb content and chemical stability of the glaze, firing temperature and quality of vitrification, food acidity, cooking temperature, contact time, repeated use, and surface wear or microfractures in the glaze. Acidic foods and prolonged heating substantially increase Pb release by promoting dissolution of poorly bound lead compounds from the ceramic surface.

In Mexico, the safety of glazed ceramic ware is regulated by NOM-231-SSA1-2016, which establishes maximum permissible limits for Pb and standardized testing procedures to evaluate their leaching from food-contact materials [23,24]. Despite these regulations, LGC continues to be an important and preventable source of Pb exposure in the Mexican population [22]. Artisanal LGC remains widely produced and used in the country because of its cultural, culinary, and economic importance, emphasizing the need for more effective strategies to promote the adoption of lead-free alternatives while preserving traditional pottery production [25].

Our findings related to home remodeling are consistent with previous studies reporting associations between renovation activities and elevated BLLs. For example, a study among recently arrived Asian women in Vancouver, Canada, reported higher BLLs associated with home renovations within the previous six months [38]. Similar associations have been described in pediatric populations, where children exposed to recent housing renovations had higher BLLs, particularly when renovations occurred within recent months [39]. Additional studies have shown that specific renovation activities, such as sanding of old paint [40], and recent home repairs [41] were associated with increased Pb exposure.

This finding is biologically plausible, as renovation activities, including sanding, cutting, and demolition, can disturb Pb-containing materials such as old paint and plumbing, releasing Pb-contaminated dust that may be inhaled or ingested [42,43]. This risk is particularly relevant in older housing, given that Pb-based paint was banned in 1978 in the United States and in 1991 in Mexico [44], suggesting that legacy materials remain an important exposure source.

The regulatory context further supports this interpretation. Although Mexico has regulations restricting Pb in paints, these do not fully align with international standards, such as the 90 ppm limit recommended by the WHO–UNEP Global Alliance to Eliminate Lead Paint [45]. In Mexico, although regulations prohibit the use of Pb compounds in the manufacture of paints and enamels for residential and institutional use, they do not establish an explicit maximum limit of 90 ppm in alignment with international standards [46]. Moreover, surveillance reports published by the non-governmental organization Casa Cem have documented that paints currently available on the Mexican market contain Pb concentrations exceeding these limits [47,48,49], highlighting an ongoing public health concern.

Living near a highway was another factor associated with elevated BLLs in our study. Previous research carried out in the Detroit Neighborhood Health Study has reported similar findings, linking residential proximity to traffic and increased metal exposure [50]. Similarly, a cohort study of Swedish schoolchildren with repeated BLLs measurements revealed that proximity to major roads significantly affected children’s BLLs during the period 1978–1987, whereas this effect was not observed thereafter, following the ban on Pb in gasoline [51].

Although the use of leaded gasoline has been banned in Mexico since 1997, historical emissions have resulted in long-term Pb contamination of soil and dust in areas near major roads [52,53]. One possible explanation for the observed association is that historical contamination from leaded gasoline may continue to have effects on the population. These legacy deposits can be resuspended into the air through traffic, wind, or construction activities, contributing to ongoing exposure [54]. Given the age distribution of our study population, it is also possible that cumulative lifetime exposure contributes to current BLLs through endogenous release from bone stores.

Some limitations should be considered. First, the study was based on a convenience sample recruited from a single community in Mexico City, which may have introduced selection bias and limits the generalizability of the findings. Participants were recruited through voluntary participation in a community-based screening program; consequently, the study population may not be representative of all women of reproductive age in Mexico City, and the findings should not be interpreted as population prevalence estimates. Second, exposure information was primarily obtained through self-reported dichotomous yes/no questions because the questionnaire was designed as a brief community-based screening instrument. Therefore, detailed information on exposure duration, frequency, timing, and intensity was not available and environmental Pb measurements in household dust, soil, paint, water, air, or LGC products were not performed. Therefore, the environmental and household variables evaluated in this study should be interpreted as associates of elevated BLLs rather than confirmed sources of exposure. Future studies integrating environmental sampling, geospatial analyses, and more detailed characterization of renovation activities with biomonitoring are needed to quantify the contribution of specific exposure pathways.

Third, BLLs were measured using capillary samples analyzed with a portable device, which may be susceptible to variability and external contamination. However, Pb determinations were performed by trained medical personnel who followed rigorously standardized protocols for proper sample collection and minimize external contamination. In addition, as part of the quality assurance procedures, BLLs were also measured in a subsample of 47 participants using inductively coupled plasma mass spectrometry (ICP-MS), the reference analytical method for Pb determination in whole blood. LeadCare^®^ II measurements showed a strong positive correlation with ICMP-MS results (Spearman’s ρ = 0.70, *p* < 0.001). In addition, Bland–Altman analysis demonstrated agreement between the two methods while also revealing a systematic positive bias, with LeadCare^®^ II tending to overestimate BLLs concentrations relative to ICP-MS by an average of 1.64 µg/dL (mean difference = −1.644 µg/dL (95% CI: −2.202 to −1.086) (Supplementary Figure S1. Importantly, no participants in the validation subsample had blood lead concentrations between 3.3 and 3.5 µg/dL. Therefore, despite the observed systematic bias, participant classification relative to the CDC blood lead reference value (≥3.5 µg/dL) was not affected.

Another limitation is the relatively small sample size, particularly the limited number of participants with elevated BLLs. This may have reduced the statistical power to detect weaker associations and contributed to wider confidence intervals for some estimates. However, several associations remained statistically significant after multivariable adjustment, supporting the potential relevance of these factors. Future studies with larger sample sizes are needed to confirm these findings and obtain more precise effect estimates.

Despite these limitations, our findings provide relevant evidence on persistent and emerging sources of Pb exposure in urban Mexican settings. These results support the need for strengthened surveillance, improved regulatory enforcement, and targeted public health interventions.

From a policy perspective, our findings highlight the importance of updating national regulations on Pb content in paints, NOM-004-SSA1-2013, to better align with international standards, as well as strengthening monitoring and enforcement mechanisms to ensure compliance [46]. Despite existing regulations, evidence suggests that Pb-containing paints may still be present in the market, underscoring the need for more effective regulatory oversight.

Furthermore, our findings are consistent with the notion that historical Pb contamination from leaded gasoline, although phased out, may continue to represent a relevant source of exposure due to its persistence in soil and dust [54]. In this context, periodic screening programs targeting vulnerable populations are essential to identify individuals with elevated BLLs and to guide timely interventions. In addition, environmental monitoring, particularly of air and soil quality in areas near high-traffic roads, should be strengthened to detect the presence of Pb and other co-occurring contaminants [55].

Finally, reported LGC use showed the greatest contribution to the explanatory performance of the multivariable model, reinforcing the need to strengthen the regulation and monitoring of these products, ensuring compliance with national standards such as NOM-231-SSA1-2016, which establishes the maximum permissible limits of soluble Pb in ceramic ware [24]. These efforts should be complemented by targeted interventions to support artisanal producers in transitioning toward lead-free glazes, while preserving cultural practices. Together, these strategies, combining regulatory strengthening, environmental monitoring, and biomonitoring, can contribute to reducing Pb exposure and improving the protection of public health in vulnerable communities.

## 5. Conclusions

This study shows that residential factors such as living near a highway and household factors, including use of LGC and recent home renovations, are associated with elevated BLLs among women of childbearing age. These findings provide updated evidence to inform epidemiological and environmental surveillance of Pb exposure in a population that has been relatively understudied in Mexico.

Our results underscore the importance of identifying and addressing potential Pb sources of exposure, as well as strengthening prevention and education strategies aimed at reducing Pb exposure in vulnerable populations.

## Figures and Tables

**Table 1:** General characteristics of the study population by capillary blood lead level (BLLs) categories (n = 158).

	Total n = 158	BLLs Categories		
		Low BLLs (<3.5 μg/dL) n = 109	Elevated BLLs (≥3.5 μg/dL) n = 49	*p*-Value
Age (years)	29.7 (6.5)	29.9 (6.4)	29.4 (6.6)	0.6412
Education				0.239
Elementary or middle school	38 (24%)	31 (28%)	7 (14%)	
High school	77 (49%)	51 (47%)	26 (53%)	
University or over	43 (27%)	27 (25%)	16 (33%)	
Marital status				0.311
Married or civil union	90 (57%)	64 (59%)	26 (53%)	
Single or divorced	68 (43%)	45 (41%)	23 (47%)	
Alcohol consumption				0.358
Yes	108 (68%)	73 (67%)	35 (71%)	
No	50 (32%)	36 (33%)	14 (29%)	
Smoking status				0.391
Yes	59 (37%)	42 (39%)	17 (35%)	
No	99 (63%)	67 (61%)	32 (65%)	
BMI				0.257
Low weight	5 (3%)	3 (3%)	2 (4%)	
Normal weight	53 (34%)	39 (36%)	14 (29%)	
Overweight	59 (37%)	39 (36%)	20 (41%)	
Obesity	41 (26%)	28 (26%)	13 (27%)	
Venous hemoglobin				0.257
Hb < 12 g/dL	27 (17%)	16 (15%)	11 (22%)	
Hb ≥ 12 g/dL	131 (83%)	93 (85%)	38 (78%)	
Nutrients, mean (IQR)				
Calcium (mg)	795.4 (539.5–1143.9)	795.4 (530.4–1063.6)	798.0 (611.2–1261.6)	0.320
Iron (mg)	13.3 (10.2–17.2)	13.3 (10.0–17.2)	13.1 (10.8–16.9)	0.653
Zinc (mg)	11.5 (8.1–16.2)	11.3 (7.7–14.8)	12.0 (8.4–16.9)	0.275

Data are presented as mean (SD), median (IQR), or n (%), as appropriate. Differences between groups were assessed using Fisher’s exact test for categorical variables and Student’s *t*-test or the Wilcoxon rank-sum test for continuous variables, as appropriate. Hemoglobin concentrations were adjusted for altitude according to the 2024 World Health Organization recommendations before anemia classification. Abbreviations: BLLs, blood lead levels; BMI, body mass index; Hb, hemoglobin; IQR, interquartile range; SD, standard deviation.

**Table 2:** Associations between potential sources of lead exposure according to blood lead level categories among women of childbearing age (n = 158).

Source of Exposure	Total (n = 158)	Low BLLs n = 109	Elevated BLLs n = 49	*p*-Value
*Environmental factors*				
Living near a pottery workshop	5 (3%)	3 (3%)	2 (4%)	0.646
Living near industrial facilities †	24 (15%)	18 (17%)	6 (12%)	0.633
Living near a highway	101 (64%)	63 (58%)	38 (78%)	**0.020**
Living near a mine/polluted river/garbage dump	77 (49%)	53 (48%)	24 (49%)	1.000
*Household factors*				
Use of lead-glazed ceramics (LGC)				**0.004**
Never used	22 (14%)	20 (18%)	2 (4%)	
Past use only	34 (22%)	26 (24%)	8 (16%)	
Current use only	18 (11%)	15 (14%)	3 (6%)	
Past and current use	84 (53%)	48 (44%)	36 (73%)	
Working on Pb-related jobs	14 (9%)	8 (7%)	6 (12%)	0.367
Living with people who have Pb-related jobs	62 (39%)	44 (40%)	18 (37%)	0.726
Living in or regularly visiting a recently remodeled home	44 (28%)	22 (20%)	22 (45%)	**0.002**
Consumption of Mexican tamarind candies	141 (89%)	99 (91%)	42 (86%)	0.406
Pica habit	18 (11%)	11 (10%)	7 (14%)	0.431
Use of Greta, Azarcon, or Navajo clay	7 (4%)	5 (5%)	2 (4%)	1.000
A personal or family history of lead poisoning	1 (1%)	1 (1%)	0 (0%)	1.000

Data are presented as n (%). Differences between BLLs categories were assessed using Fisher’s exact test. Abbreviations: Pb, lead; BLLs, blood lead levels; LGC, lead-glazed ceramics. Statistical significance was defined as *p* < 0.05. † Industrial facilities included radiator workshops, ironworks, laundries, paper mills, textile factories, and printing plants. For dichotomous variables, only the presence of the characteristic (“Yes”) is shown.

**Table 3:** Crude and adjusted odds ratios for factors associated with elevated blood lead levels (BLLs) among women of childbearing age (n = 158).

	Crude OR			Adjusted OR (Model 1)			Adjusted OR (Model 2)		
	OR	95% CI	*p*-Value	OR	95% CI	*p*-Value	OR	95% CI	*p*-Value
*Environmental factors*									
Living near a highway	2.52	(1.17–5.45)	**0.019**	2.39	(1.09–5.23)	**0.030**	2.41	(1.07–5.48)	**0.034**
*Household factors*									
Use of lead-glazed ceramics (LGC)									
Past use only	3.08	(0.59–16.11)	0.183	3.10	(0.59–16.11)	0.186	3.87	(0.68–22.07)	0.127
Current use only	2.00	(0.30–13.51)	0.477	1.79	(0.26–12.23)	0.554	2.34	(0.31–17.68)	0.409
Past and current use	7.49	(1.65–34.17)	**0.009**	6.93	(1.50–32.01)	**0.013**	9.43	(1.85–48.15)	**0.007**
Living in or regularly visiting a recently remodeled home	3.22	(1.55–6.70)	**0.002**	2.91	(1.37–6.21)	**0.006**	3.21	(1.45–7.11)	**0.004**

Odds ratios (ORs) and 95% confidence intervals (CIs) were estimated using logistic regression models. Model 1 was adjusted for age, education, and marital status. Model 2 was additionally adjusted for calcium intake, zinc intake, smoking status, body mass index (BMI), and altitude-adjusted hemoglobin. For dichotomous variables, “No” was used as the reference category. For use of lead-glazed ceramics, “Never used” was the reference category. Abbreviations: Pb, lead; BLLs, blood lead levels; LGC, lead-glazed ceramics.

**Table 4:** Relative contribution of lead exposure sources to the pseudo-R^2^ of the logistic regression model based on Shapley decomposition.

Lead Exposure Source	Relative Contribution (%)
Use of lead-glazed ceramics	47.46
Living in or regularly visiting a home recently remodeled	31.78
Living near a highway	20.76
Total contribution	100.00

Percentages represent the relative contribution of each exposure source to the model pseudo-R^2^ and do not imply causality. Lead-glazed ceramic use was modeled as a four-category variable and evaluated jointly as a single exposure factor.

## Data Availability

The data presented in this study are available from the corresponding author upon reasonable request. The data are not publicly available because they contain information that could compromise participant privacy.

## Supplementary Materials

The following supporting information can be downloaded at https://www.mdpi.com/article/10.3390/toxics14080695/s1, Figure S1: Comparison between LeadCare^®^ II and ICP-MS measurements of blood lead levels in a validation subsample (n = 47); Table S1: Crude and adjusted odds ratios for factors associated with elevated blood lead levels (BLLs) among women of childbearing age (n = 158).
