# Supplementary material for: Environmental and Household Sources Associated with Elevated Blood Lead Levels Among Mexican Women of Childbearing Age: A Community-Based Screening Study
Source: Toxics. 2026 Aug 6;14(8):695. doi: 10.3390/toxics14080695 (PMC13517566; doi:10.3390/toxics14080695)
Supplement: Supplementary file 1 [file toxics-14-00695-s001.zip › toxics-4358563-supplementary.pdf]

Supplementary Material

Supplementary Table S1. Crude and adjusted odds ratios for factors associated with elevated blood lead levels (BLL) among women of childbearing age (n = 158).

	Crude OR			Model 1			Model 2		
	OR	95% CI	p-value	OR	95% CI	p-value	OR	95% CI	p-value
**Environmental factors**									
Living near a pottery workshop	1.50	(0.24-9.30)	0.66	1.72	(0.26-11.18)	0.57	1.81	(0.27-12.02)	0.54
Living near industrial facilities†	0.71	(0.26-1.90)	0.49	0.69	(0.25-1.90)	0.47	0.82	(0.28-2.38)	0.71
Living near a mine/polluted river/garbage dump	1.01	(0.52-1.99)	0.97	1.15	(0.57-2.30)	0.69	1.26	(0.61-2.59)	0.54
**Household factors**									
Working on Pb-related jobs	1.76	(0.58-5.38)	0.32	2.04	(0.64-6.55)	0.23	2.70	(0.79-9.20)	0.11
Living with people who have Pb-related jobs	0.86	(0.43-1.72)	0.67	0.87	(0.43-1.76)	0.69	0.89	(0.43-1.86)	0.76
Consumption of Mexican tamarind candies	0.61	(0.22-1.70)	0.34	0.50	(0.17-1.47)	0.21	0.49	(0.16-1.51)	0.21
Pica habit	1.48	(0.54-4.09)	0.45	1.33	(0.47-3.79)	0.59	1.42	(0.49-4.16)	0.52
Use of Greta, Azarcon, or Navajo clay	0.89	(0.17-4.73)	0.89	0.87	(0.15-4.88)	0.87	0.88	(0.15-5.19)	0.89
A personal or family history of lead poisoning		*			*			*	

Odds ratios (ORs) and 95% confidence intervals (95% CIs) were estimated using logistic regression models. Model 1 was adjusted for age, education, and marital status. Model 2 was additionally adjusted for calcium intake, zinc intake, smoking status, body mass index (BMI), and altitude-adjusted hemoglobin concentration. For dichotomous variables, the reference category was women who answered "No".†Industrial facilities included radiator workshops, ironworks, laundries, paper mills, textile factories, and printing plants. *The OR was not estimated due to perfect prediction between the variables

Supplementary Figure S1. Comparison between LeadCare® II and ICP-MS measurements of blood lead levels in a validation subsample (n = 47).

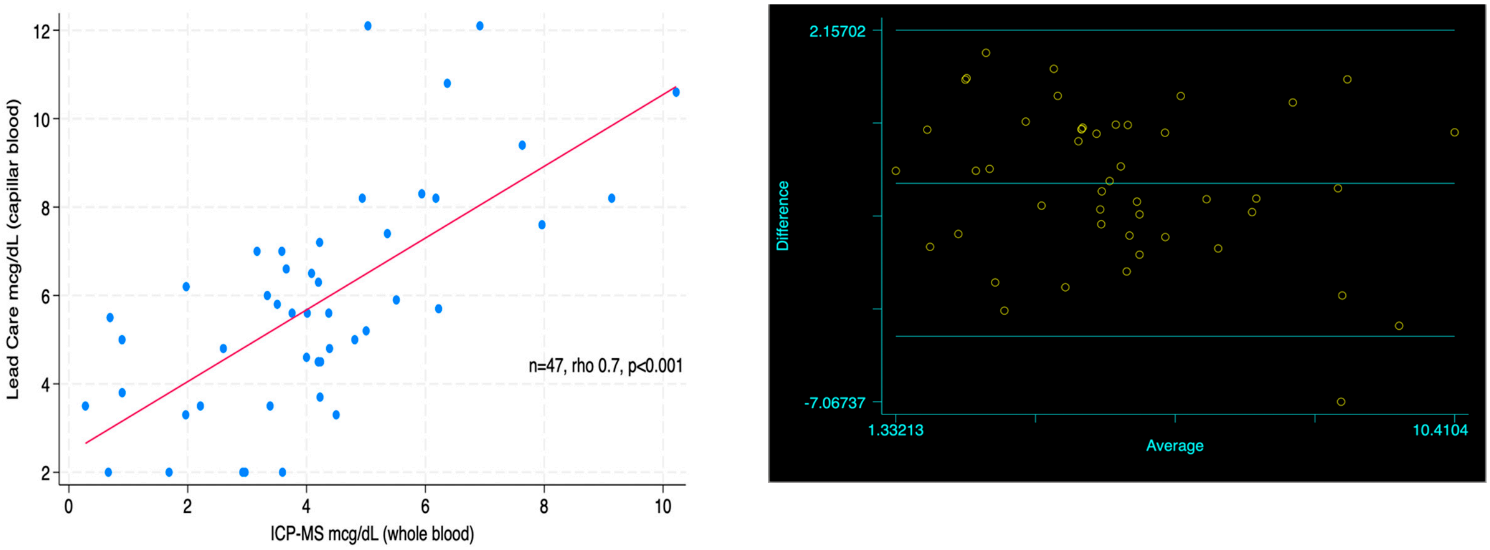

(A) Scatter plot showing the association between capillary blood lead levels measured using the LeadCare® II analyzer and whole-blood lead concentrations measured by inductively coupled plasma mass spectrometry (ICP-MS), the reference analytical method. LeadCare® II measurements were strongly correlated with ICP-MS measurements (Spearman's $\rho = 0.70$, $p < 0.001$).

(B) Bland–Altman plot showing the agreement between LeadCare® II and ICP-MS measurements. The mean difference (ICP-MS − LeadCare® II) was **−1.64 µg/dL** (95% CI: **−2.20 to −1.09**), indicating that LeadCare® II yielded, on average, higher blood lead concentrations than ICP-MS. The 95% limits of agreement ranged from **−5.45 to 2.16 µg/dL**. These analyses were performed as part of the study quality assurance procedures to evaluate the performance of the portable LeadCare® II analyzer relative to the reference method.
